# Collective Motion as an Ultimate Effect in Crowded Selfish Herds

**DOI:** 10.1038/s41598-019-43179-6

**Published:** 2019-04-29

**Authors:** Wen-Chi Yang, Thomas Schmickl

**Affiliations:** 1 Department of Computer Science and Technology, Henan Institute of Technology, Xinxiang, 453003 China; 2 Artificial Life Lab of the Institute of Biology, Karl-Franzens University of Graz, A-8010 Graz, Austria

**Keywords:** Behavioural ecology, Ecological modelling, Evolutionary theory

## Abstract

The selfish herd hypothesis explains how social prey can assemble cohesive groups for maximising individual fitness. However, previous models often abstracted away the physical manifestation of the focal animals such that the influence of getting stuck in a crowded herd on individual adaptation was less intensively investigated. Here, we propose an evolutionary model to simulate the adaptation of egoistic social prey to predation given that individual mobility is strictly restrained by the presence of other conspecifics. In our simulated evolutionary races, agents were set to either be confined by neighbours or move to empty cells on the lattice, and the behavioural traits of those less exposed were selected and inherited. Our analyses show that under this crowded environment, cohesive and steady herds were consistently replaced by morphing and moving aggregates via the attempt of border agents to share predation risk with the inner members. This kind of collective motion emerges purely from the competition among selfish individuals regardless of any group benefit. Our findings reveal that including the crowding effect with the selfish herd scenario permits additional diversity in the predicted outcomes and imply that a wider set of collective animal behaviours are explainable purely by individual-level selection.

## Introduction

**Figure 1:**
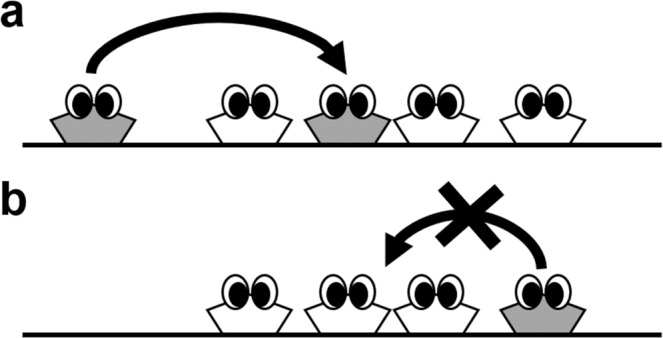
The selfish herd scenario with the crowding effect. A selfish herd emerges due to the border individuals moving towards the secure central positions. Hence, the group should finally become too crowded for the border individuals to enter freely.

The selfish herd hypothesis^1^ has long been adopted to explain the flocking behaviour of gregarious animals under predation. This hypothesis suggests that when prey individuals face predatory threats in open space, apart from the functional advantages of aggregating, e.g., the confusion effect^2^ and information transfer effect^3^, the benefit of being covered and protected by other individuals is sufficient to drive prey into dense herds (Fig. 1a). This dynamic has been observed in many group-living organisms. The most popular examples are various species of schooling fish, which assemble compact groups under the risk of predation^4–6^. Fieldwork studies have also linked specific bird flocks into the selfish herd^7–9^. Additionally, vigilant fiddler crabs^10^, seals^11^ and sheep^12^ have been reported to reduce individual distances within a group.

Several modelling studies have examined how and why selfish herds emerge from the interaction of prey individuals. For example, instead of the attraction to the nearest neighbour^1^, reasonably complicated movement rules based on group densities and individual positions have been demonstrated as necessary for the emergence of large groups in two-dimensional space, the environment of most terrestrial animals^13–17^. In consideration of the ultimate reasons, a growing number of simulations have demonstrated that under the selfish herd scenario, the evolution of prey agents’ movement strategies can result in stable states where selfish agents assemble large and dense groups^18–22^. These theoretical studies indicate that from both proximate and ultimate aspects, the selfish herd scenario would emerge and persist during evolution.

Nevertheless, the specific impact of crowding effects on aggregating was less considered and examined in related works. For example, a prey agent in a selfish herd model was often treated as a (volume-less) point^13–19^ or assigned a constant speed^18–22^, despite that limited mobility due to the physical embodiment of agents has been an important topic for swarm robotic algorithms^23,24^ and human crowd simulations^25,26^. The simplifications in traditional selfish herd models may prevent their outputs from explaining real collective behaviours in more detail. For example, since prey prefers the group interior, a herd must increase its density with time, and finally become too crowded for latecomers to enter freely, simply because of the lack of space (Fig. 1b). In this situation, many movement rules and emergent patterns previously demonstrated would result in the overlap of individual private space or individual bodies, and hence would be physically impossible for real animals.

Therefore, we aimed to revisit the selfish herd scenario by further considering the effect of crowdedness. Specifically, the limited individual sensing range adopted by most of the related models has captured a typical condition in a dense group^27^. The collision-repulsion^20^ or collision-penalty^21,22^ settings have also been popular, which implicitly account for the individual private zones or body extensions. However, the influence of limited mobility in crowded selfish herds has been studied less. Comparatively, this research is more popular in both empirical and theoretical studies in human crowds, e.g., pedestrian behaviours^25,26^ or traffic flows^28,29^. These studies have revealed that the property of crowdedness significantly shapes various collective human patterns such as crowd turbulence and stop-and-go waves (phantom traffic jams). Hence, it is reasonable to assume that physical crowdedness should also affect the adaptation of social animals.

In this study, we created an evolutionary selfish herd model based on the lattice gas framework^28–30^ to investigate how the property of crowdedness can affect the evolution of movement strategies under the selfish herd scenario. Similar to previous selfish herd models^18–20^, the intraspecific competition in a prey population was highlighted in the proposed model, in which prey agents that had been less covered and protected by their neighbours were assumed to be predated and were replaced by new agents. These new agents were offspring of the survivors and hence inherited their parents’ beneficial behavioural traits (see Methods, Evolutionary Procedure). This typical simulated evolution abstracted away predator-prey relations and interspecific competitions so that the selected behavioural genotypes, which were two-layer artificial neural networks in our model (see Methods, Behavioural Genotypes), maximised short-term individual fitness, i.e., selfishness.

To simulate individual movements under the crowding effect, we implemented an open space for the agents’ interaction using a two-dimensional lattice and determined that movement of an agent is valid only if the target cell (one of the four adjacent cells) is empty; otherwise, an agent remains stuck near its neighbours (see Methods, Interaction Rules). Compared with previous models^18–22^, an agent in the proposed model must make movement decisions under strictly restrained mobility without the option of passing through others and entering the group centre. Our goal was to examine the selfish movement rules selected under a crowded environment.

In addition to demonstrating the emergent patterns in a crowded situation, we analysed both proximate and ultimate mechanisms that impel selfish agents into these formations. We also considered the impact of crowdedness among different animal types, in which the degrees of mobility inside a dense group could vary from fully restrained to completely unhindered. Thus, we may further disclose and highlight the effect of crowdedness in collective animal behaviours.

## Results

### Evolutionary Trajectory at the Population Level

The adaptation of prey agents to predation risk in the model was simulated for 500 evolutionary runs. Each evolutionary run consisted of 3,000 generations, as reproductive cycles of selection and adaptation in an agent population, and each generation consisted of 2,000 time steps, as the rounds of agents’ movements on the lattice. To quantify the features of the agents’ behaviours, we defined two metrics: the neighbourhood score (${S}_{i}^{N}$, the expected number of occupied cells in an agent *i*’s neighbourhood domain per time step) and the mobility score (${S}_{i}^{M}$, the expected frequency that an agent *i* moves to a vacant adjacent cell) (Eqs (2) and (3) in Methods). The neighbourhood domain is different from the movement range at a time step, i.e., the four adjacent cells^30^. This area is associated to the limited domain of danger^16^ and was set to the Moore neighbourhood with radius $r=1$ in the present work (see Methods, Parameter Scans). Hence, in the following analyses, the maximum neighbourhood score of an agent is 8 when all cells in the neighbourhood domain stay occupied with time steps.

**Figure 2:**
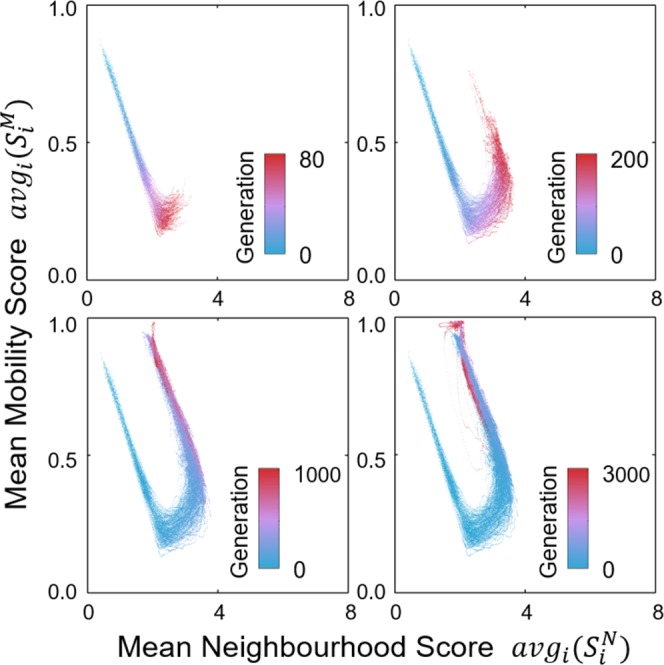
Evolutionary trajectories from 500 independent evolutionary runs, coloured based on the timing of generations in the evolutionary courses (blue: early, red: late). The x-axis is the mean neighbourhood score in a generation, i.e., the average number of neighbours per agent per time step. The y-axis is the mean mobility score in a generation, i.e., the frequency of changing the occupied cells per agent per time step. Based on these two indexes, an evolutionary sequence can be divided into three phase transitions between the 0^th^, 80^th^, 200^th^, and 1,000^th^ generations. The metrics are defined in the Methods section.

As shown in Fig. 2, according to the mean neighbourhood scores and the mean mobility scores of different generations, the evolutionary runs were qualitatively highly consistent in their outcomes, in which the three major phase transitions were observed between the 0^th^, 80^th^, 200^th^, and 1,000^th^ generations. Hence, we treated the four generations as representative evolutionary epochs in the following analyses.

**Figure 3:**
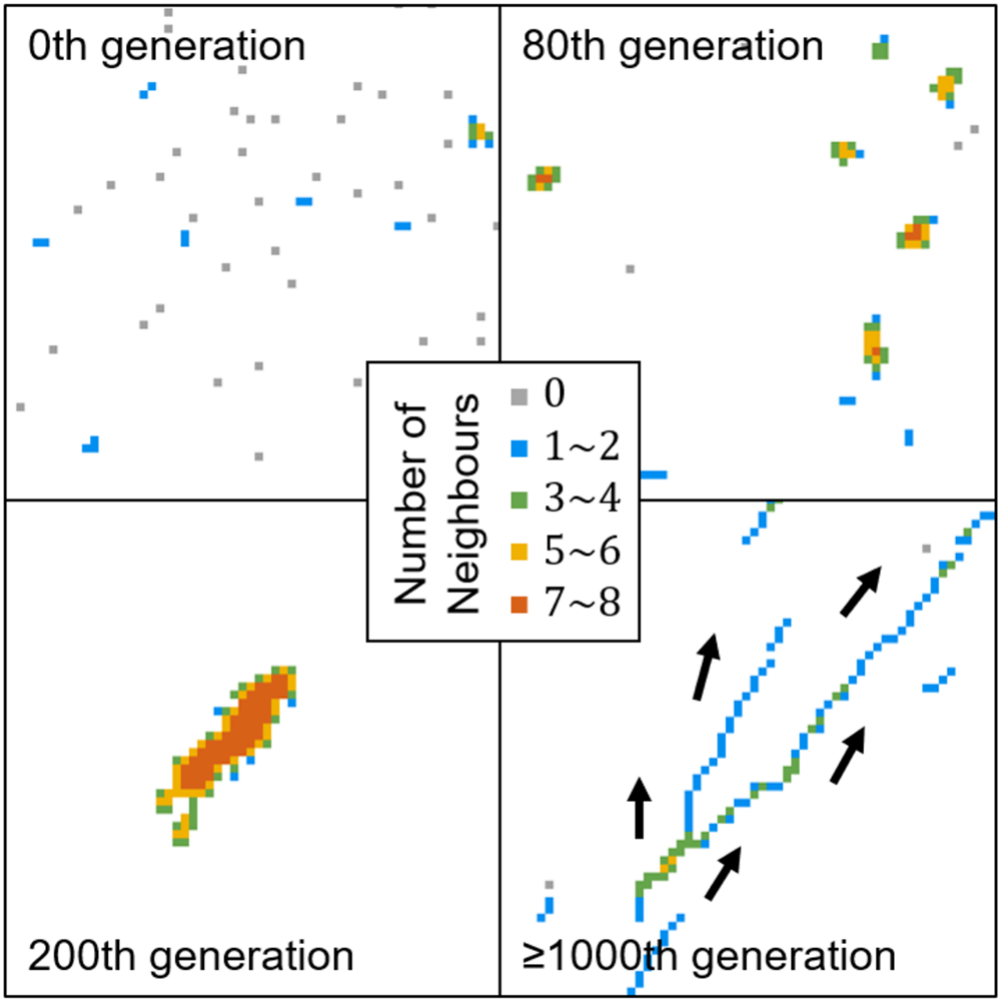
The emergent patterns in the four evolutionary epochs. For improved visualisation, only a quarter of the lattice is displayed. In the last subfigure, the arrows indicate the heading directions of prey agents, similar to the collective motion of gnus or antelopes (see Supplementary Video S1).

As quantified in Fig. 2 and visualised in Fig. 3, the emergent patterns are different in the four evolutionary epochs. In the initial generation, prey agents moved randomly and hence most individuals were alone (Fig. 3, upper left). After the 80^th^ generation, prey agents formed numerous groups of various sizes (Fig. 3, upper right). After the 200^th^ generation, few stationary herds were assembled, in which the inner agents were surrounded by 7 or 8 neighbours and safer than the outer agents (Fig. 3, lower left). After the 1,000^th^ generation, elongated and drifting herds appeared consistently, and most agents were equally surrounded by approximately 2 neighbours (Fig. 3, lower right).

### Movement Rules in Evolutionary Epochs

**Figure 4:**
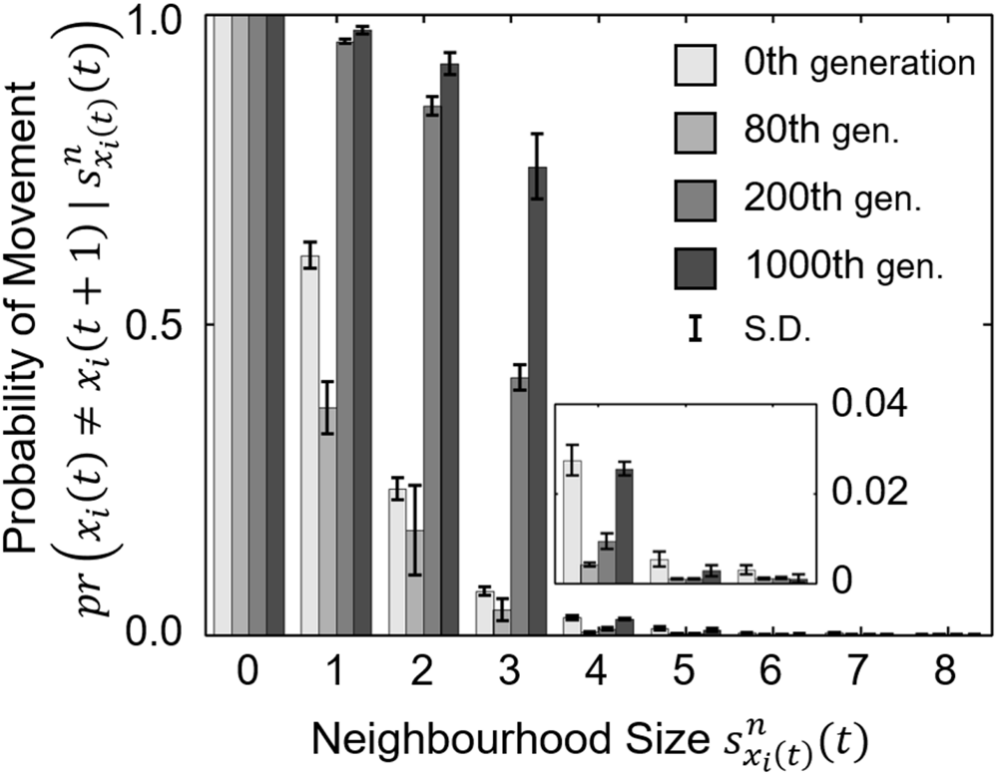
The conditional probability of an agent leaving the present cell depending on the neighbourhood size. In each of the four evolutionary epochs, we separated the frequency of moving to an empty adjacent cell (y-axis) by the number of neighbours (x-axis). Agents in the 80^th^ generation evolved to stay with neighbours under any condition, but after the 200^th^ generation, agents evolved to leave few neighbours. The measurement in the 0^th^ generation provides the results of a null model. The small graph is a zoomed-in display of the measured probabilities given neighbourhood sizes of 4–6.

To discriminate between the individual movement rules in the four evolutionary epochs that caused the emergence of different patterns, we measured the conditional probabilities of moving towards a vacant cell given different neighbourhood sizes of the present location (Eq. (4) in Methods). For example, this probability must be 1.0 when agents are in isolation and 0.0 when agents are surrounded by 8 neighbours in its neighbourhood domain. The error bars displayed in Fig. 4 show that the variation of individual movement rules is small in every evolutionary epoch and hence these measurements are sufficient to represent the features of individual tactics.

Compared with prey agents in the initial generation, those in the 80^th^ generation evolved to stay with neighbours much more frequently, regardless of how many neighbours it had (Fig. 4, 80^th^ generation). This movement rule is hereafter termed the *coward strategy*, as agents retained close proximity to one another and avoided being alone. When most agents had exhibited the *coward strategy*, a population’s spatial configuration self-organised into a set of many small groups within the environment (Fig. 3, upper right).

In the 200^th^ generation, prey agents displayed a movement rule that essentially differed from the *coward strategy*: the agents increased the frequency of leaving their present cells when they had less than 4 neighbours (Fig. 4, 200^th^ generation). Consequently, prey agents looked for sufficiently large groups to join and assembled huge and stationary herds (Fig. 3, lower left). As shown in Fig. 2, this adaptation increases both the mean mobility score (due to the departure of agents from small groups) and the mean neighbourhood score (because agents only stay in large groups) of a population. To highlight the emergence of a searching behaviour for more beneficial positions, we termed this behavioural rule the *explorer strategy*.

After the 1,000^th^ generation, prey agents exhibited a tendency to leave their present cells even when they had more than 3 neighbours (Fig. 4, 1,000^th^ generation). This result suggests that agents evolved to stay only when they were in the group interior, thereby maximising their selfishness during the evolutionary course. Triggered by the high mobility of border individuals, prey agents formed elongated herds and exhibited collective motion. This emergent phenomenon decreased the mean neighbourhood size in the population to a great extent (Fig. 2, lower left). To highlight the avoidance of border positions that caused the disappearance of the group interior, this tactic was termed the *dodger strategy*.

**Figure 5:**
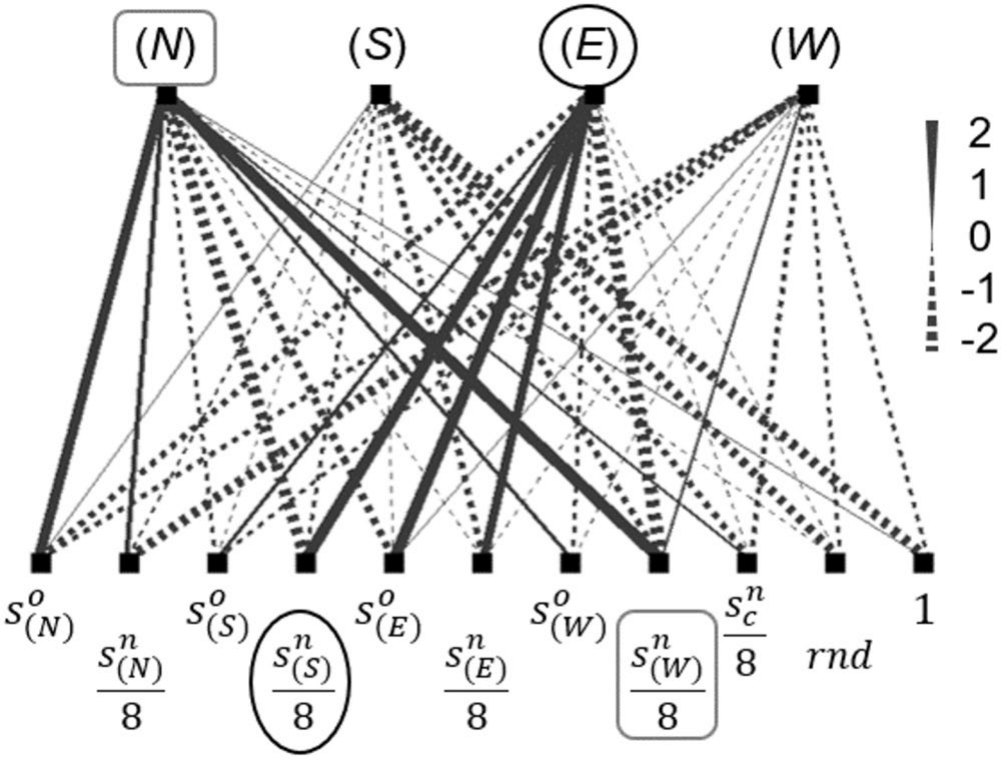
An exemplary network topology that moves an agent in an evolved *dodger strategy*, i.e., shifting along the herd edge to expose inner neighbours. The network acquires the occupancy states (whether a cell is occupied) and neighbourhood sizes (the number of Moore neighbourhood agents) of the four adjacent cells and triggers the heading direction with the maximal output value. Specifically, when the western cell has a large neighbourhood size, the preference towards moving to the northern cell is enhanced (the grey squares). The southern cell’s neighbourhood size also positively affects the movement towards the east (the black circles).

To further explain the characteristics of the movement rules leading to the *dodger strategy*, Fig. 5 displays the network topology of the fittest agent in the 3,000^th^ generation from the simulation that Fig. 3 visualised. It shows the agent shifted along the group edge, rather than leaving in isolation, once its western or southern cells had a large neighbourhood size. Notably, the agent reacted differently to its eastern or northern neighbours (Fig. 5). In other words, the *dodger strategy* exploited information about the relative positions of neighbours, and a particular adaptive response was reinforced with generations stochastically. This property was less utilised by the two transitional strategies.

### The Dynamics of Adaptation During Evolution

**Figure 6:**
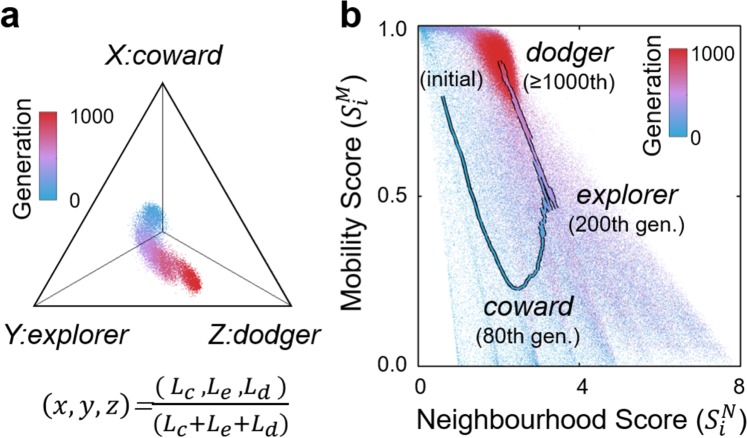
Adaptive dynamic of strategies and risk distribution of individuals. In panel (a) the adaptive dynamic was measured based on the likelihood values for the three corresponding strategies, i.e., *L*_*c*_, *L*_*e*_ and *L*_*d*_. It shows the individual neural networks, as the behavioural genotypes, were relatively homogeneous in each generation. In panel (b) the curve is the mean mobility score along the mean neighbourhood score and the dots display all individuals’ associated values, showing that the variance within individual observations was reduced with succeeding strategies. In both panels, generations are indexed by colour (blue: early, red: late).

We further analysed the deviation of individual traits at both the genotype and phenotype levels to identify the underlying mechanisms of the phase transitions. At the genotype level, we located each individual tactic based on the associated likelihood values between its neural network and the three chosen network topologies, which were the average network topologies at the three evolutionary epochs, i.e., the 80^th^, 200^th^ and 1,000^th^ generations, to represent general structures of the three significant strategies during evolution. Thus, we positioned each agent *i* in three-dimensional space by the three likelihood values: $L(i{,}^{{\rm{^{\prime} }}}cowar{d}^{{\rm{^{\prime} }}})$, $L(i{,}^{{\rm{^{\prime} }}}explore{r}^{{\rm{^{\prime} }}})$ and $L(i{,}^{{\rm{^{\prime} }}}dodge{r}^{{\rm{^{\prime} }}})$ (Eq. (5) in Methods). Figure 6a shows that during the transition from the *coward strategy* via the *explorer strategy* to the *dodger strategy*, the positions of individual traits aggregate in a dense band, which implies that all collective behaviours, including the polarised collective motion, were driven by relatively homogeneous agents without the existence of social roles or evolutionary branches. This was a general phenomenon in our evolutionary runs inasmuch as the simulation outputs were consistent (Fig. 2).

To capture the underlying mechanisms that caused the sequential emergence of the three significant strategies, we measured and displayed the distributions of all individual’s mobility scores (Eq. (3) in Methods) and neighbourhood scores (Eq. (2) in Methods) within and across generations (Fig. 6b). The blue curve in Fig. 6b shows that, by adopting the *coward strategy* and aggregating locally, agents on average had more neighbours than in the initial generation. Hence, the trajectory indicated that the *coward strategy* developed in early generations through the elimination of solitarily moving agents and the removal of their behavioural traits from the gene pool.

Although agents adopted the same *coward strategy* after approximately the 80^th^ generation (Fig. 4, 80^th^ generation), the different sizes of emergent local groups led to an uneven distribution of individual neighbourhood scores within a prey population, which varied from 1 to 8 neighbours per individual per time step (blue dots in Fig. 6b). Thus, agents which had chosen to stay in pairs or small groups were expected to be predated upon and replaced by the offspring agents from those that had evolved to depart from cells surrounded by few neighbours, i.e., the *explorer strategy*, as the measured movement rule in the 200^th^ generation in Fig. 4. After most agents had already adopted the *explorer strategy*, the distribution of neighbourhood scores among prey agents appeared with reduced variance and had between 3 and 8 neighbours per time step (purple dots in Fig. 6b).

Further along the evolutionary trajectory, prey agents evolved to abandon cells with 3, 4, or even 5 neighbours (Fig. 4, 1,000^th^ generation) to prevent themselves from being most at risk within the population. Consequently, agents evolved to stay in the group interior to shield themselves from all sides. Because every group must contain an outer region with exposed agents, a collective movement emerged from the continuous departure of group members that were at the border positions. In this situation, individual neighbourhood scores decreased again and converged to a value of approximately 2 neighbours per time step (red dots in Fig. 6b), which reflects the unavailability of suitable central positions in prey herds (Fig. 3, lower right). This state was demonstrated evolutionarily stable since no mutant strategies were able to perturb it for sufficiently many generations (Fig. 2, lower right).

### Comparative Experiments

The evolutionary replacement of the *explorer strategy* by the *dodger strategy* was analysed as a consequence when the latecomers were restrained from entering the interior of a crowded group. This transit can be, however, somehow feasible in particular populations, like animals with soft or squeezable bodies. In this case, it is unclear whether the *dodger strategy* is still evolutionarily stable. To cover all configurations that could be found in nature, we designed a comparative variation of the model, in which a border agent was allowed to squeeze itself into an occupied cell by swapping the positions between itself and its target with the probability *p*_*transit*_. Hence, in simulations with ${p}_{transit} > 0$, a border agent is possible to squeeze itself into the herd by pushing the inner neighbours to the outer and more exposed areas.

**Figure 7:**
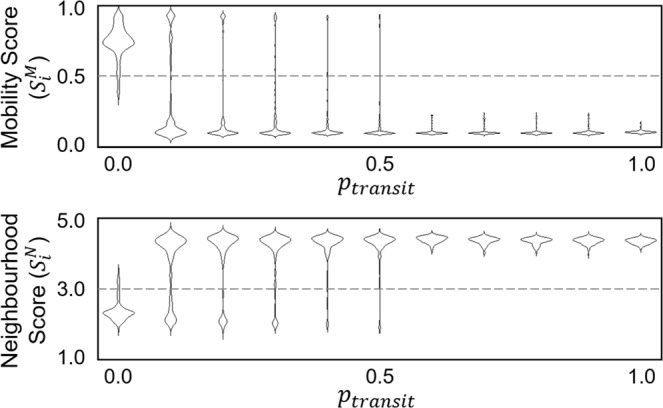
The two main evolutionarily stable states under different *p*_*transit*_ configurations. The violin plots show when ${p}_{transit} < 0.5$, the collective motion pattern was stable and attainable in evolution. In contrast, when ${p}_{transit} > 0.0$, a state of stationary herds, in which agents exhibited low mobility scores and high neighbourhood scores, was also evolutionarily stable. All *p*_*transit*_ values were sampled at an interval of 0.1 units from 0.0 to 1.0. In each configuration, we performed 100 evolutionary runs to collect the evolved states in the 3,000^th^ generation.

Figure 7 displays the simulation outputs from full blocking (${p}_{transit}=0$, as the model in our previous experiments) through hindered transit ($0 < {p}_{transit} < 1$) to unhindered transit (${p}_{transit}=1$, as a reference to traditional selfish herd models). When $0 < {p}_{transit} < 0.5$, the hourglass-shaped violin plots in Fig. 7 show that an evolutionary simulation stochastically reached one of the following two evolutionarily stable states. One reachable state was characterised by a collective motion pattern in which agents moved to vacant cells frequently in association with 2 other neighbours, which was demonstrated in our previous experiments. The other stable state was characterised by the emergence of cohesive and stationary herds in which agents stayed with mostly 4–5 neighbours and avoided moving to the exposed positions (see Supplementary Video S2), which became the only evolutionarily stable state after ${p}_{transit}\ge 0.5$. It is also shown in Fig. 7 that no other states, e.g., the coexistence of stationary and mobile herds, were stable in evolution substantially.

**Figure 8:**
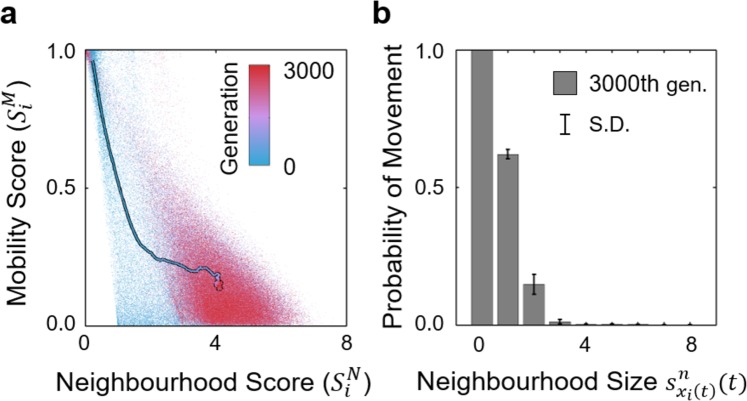
Evolutionary trajectory without the crowding effect. Panel (a) shows that compared with Fig. 6b, when agents adopted the *explorer strategy*, the distribution of individual neighbourhood scores converged, and the phase transition ended. In panel (b) the conditional probabilities of leaving the present cell to an empty one in the 3,000^th^ generation show that agents adopted the *explorer strategy* and left small groups.

To explain why stationary herds can evolve given ${p}_{transit} > 0.0$, we ran an evolutionary simulation when ${p}_{transit}=1.0$ for example, where the crowding effect was eliminated as in classic selfish herd models. Figure 8a shows the evolutionary trajectory was similar to that when ${p}_{transit}=0.0$ (the case of full blocking) until prey agents adopted the *explorer strategy* (Fig. 8b). Compared with Fig. 6b, when agents adopted the *explorer strategy*, the neighbourhood scores between individuals were highly converged in this experiment (red dots in Fig. 8a) because border agents had no resistance to enter the interior. This situation led to an equal survival probability among the group members and curbed the transition towards the *dodger strategy*, i.e., the departure of border agents.

The comparative experiments demonstrated that when individual mobility was less restrained by physical crowdedness, the *explorer strategy* and its emergent cohesive herds were evolutionarily stable. In contrast, the limited mobility in a crowded herd shaped the evolutionary trajectory towards the *dodger strategy* and its emergent collective motion. There was a wide parameter range in which both evolutionarily stable states were attainable such that simulation runs reached any of the two states stochastically.

## Discussion

A cohesive herd in which prey individuals squeeze into the central positions has long been the implication of the selfish herd scenario^1^, and the exclusion of crowding effects was considered a reasonable simplification. Nevertheless, due to the limited mobility from body extensions or private zones, the attempt to squeeze into a crowded group can fail, resulting in the outer prey individuals remaining at the dangerous border. We have demonstrated that this crowded situation drove the whole system towards a new evolutionary equilibrium, where a collective motion emerged from the spontaneous movement of the exposed border agents. This adaptation for individual short-term fitness even reduced the whole population’s survival fitness in our model. The findings imply individual-level selection, as individuals competing for secure placements with their conspecifics^1^, can explain a more diverse set of collective animal behaviours if crowding issues are considered more specifically.

The three significant strategies observed in the evolutionary epochs of our model show a strong analogy to previous modelling studies. The *coward strategy* is equivalent to the movement rule designed in the original selfish herd model^1^, i.e., movement towards the nearest neighbours. As indicated by previous modelling^13^ and empirical^31^ studies, the *coward strategy* does not cause the emergence of large herds in two-dimensional space. Instead, a density-dependent movement rule is required^14,15^. This is the *explorer strategy* observed in our model, by adopting which tactic prey gave up small local groups and searched for bigger ones. Lastly, we have shown that with the consideration of crowdedness, the *dodger strategy* evolved and led to the emergence of collective motion. The physical mechanism of this emergence has been demonstrated as *symmetry breaking behaviour*^32^, which indicates the phenomenon that agents looking for increasingly large herds eventually causes the collapse of steady herds. These compatible outputs may enhance the generality and validity of our findings.

From a modelling aspect, previous evolutionary models that assigned agents a constant speed and a predesignated alignment behaviour were criticised for the implicit causation between the settings and the emergent collective motion^33,34^. In our model, agents were extremely likely to stop and be stuck due to crowding. Even under this environment, the collective motion evolved consistently, with clear mechanisms at both proximate and ultimate aspects. Our results support and deepen the understanding of several related models. For example, instead of a constant speed, an alignment behaviour, or additional anti-predator functions, the key factor for the emergence of collective motion in several evolutionary models^20–22^ would be their collision-repulsion or collision-penalty settings, which may indirectly create an environment where ${p}_{transit} < 0.5$, such that entering the group centre is difficult. In contrast, models that excluded the crowding effect and evolved prey agents into stationary herds^18,19^ can be compared to the case when ${p}_{transit} > 0.5$. Moreover, the evolved *dodger strategy* in our model indicates that the spontaneous departure of agents is an important model design, which setting was only apparent in few modelling studies^19,32^.

The collective motion patterns in group-living animals have been explained with various aspects. For the proximate reasoning, different self-organising mechanisms, from identical conspecifics^35,36^ to diverse individuals^37,38^, have been proposed and well-studied. For the ultimate reasoning, evolutionary trade-offs^39,40^ and anti-predator benefits^2,3^ have been widely accepted to explain why animal aggregation moves in coordination. Specific mechanisms have also been documented, such as the cannibalism of locusts (individuals pursue the front neighbours and escape from the behind ones)^41^ and stampedes of people (individuals are pushed to move)^25^. The *dodger strategy* reported in the present work, which involves the initiative of border individuals to leave and expose inner neighbours, is an original attempt to explain animal movement coordination based on the selfish herd scenario, i.e., intraspecific competition, with additional consideration of the crowding effect.

Often, the coordinated movements of many gregarious organisms are discussed as being shaped by the environment, e.g., lobster queuing march^42^, or as being influenced by the group benefit, e.g., fish schools and bird flocks^2,3^. However, in our model, similar structures emerged from pure egoistic optimisation without any physical environmental factors or any interspecific competition. Furthermore, we chose a random allocation scheme at the beginning of every generation and designed the mating mechanism without niching or speciation in order to avoid effects of kin selection or group selection. In this model configuration, we demonstrated that collective motion still emerged as a result of selection based on individual short-term fitness. The study highlights that animal aggregation dynamics can be driven by a simple selective force with impact of hidden factors such as crowdedness and may contribute to a novel perspective on the evolution of collective animal behaviour.

## Methods

### Model Framework

Given that individual mobility is restrained by the existence of neighbours, beneficial movement strategies can become very sophisticated. We proposed an evolutionary lattice gas model^30^ where the location of a prey agent was discretised into cells on a lattice such that crowdedness can be implemented by forbidding agents from moving into the occupied cells. Thus, we abstracted the body or territory of a prey individual into the area of a cell on the lattice. This structure effectively reduces the computational complexity and maintains the model’s robustness.

As an overview of the evolutionary model, we permitted 200 prey agents to interact with one another on a 120 × 120 wrapped-around lattice in every generation. These agents executed their movements iteratively by seeking a safe place to survive predation under a crowded situation. After sufficiently large time steps for their interaction, agents with the lowest number of neighbours within a generation were assumed to be predated and were replaced by the offspring agents of the survivors. In the experiments, each evolutionary run consisted of 3,000 generations (reproductive cycles), and each generation consisted of 2,000 time steps (movement rounds).

### Interaction Rules

At the beginning of every generation, prey agents were placed on the lattice at random without any overlap between their cells, as the projected areas of their bodies or territories on the surface. This starting condition simulates the loose aggregates of group-living animals before the onset of predatory hazards^4–12^. In every generation, we set 2,000 time steps for the interaction of vigilant agents in predation. At each time step *t*, all agents move once in a sequential order slightly different from that of the previous time step, so that the design simulates the iterative response between neighbouring individuals and avoids the bias from a fixed order^43,44^. Each agent decides to move towards one of the four adjacent cells through the function of its genotype (a two-layer artificial neural network) and local information, i.e., the occupancy states and the neighbourhood sizes of its four adjacent cells. If the target has already been occupied by another individual, the agent must remain in the same cell due to the constraint of crowdedness in our model.

The neighbourhood information was updated as follows in an asynchronous way. At any time step *t*, each of the 120 × 120 cells can be either vacant or occupied by one agent, recorded by its occupancy state ${s}_{c}^{o}(t)=0$ and ${s}_{c}^{o}(t)=1$, respectively, where $c=(x,y)$ denotes the cell at row *x* and column *y* on the lattice. Associated with the limited domain of danger^16^, we defined the neighbourhood size of cell *c* at time step *t*, ${s}_{c}^{n}(t)$, as the summation of the occupancy states of the cells in its neighbourhood domain, set by the Moore neighbourhood with radius $r=1$ in the present work (see Methods, Parameter Scans):

1 $${s}_{c}^{n}(t)=\sum _{j\in {N}_{c}}\,{s}_{j}^{o}(t),$$

where *N*_*c*_ denotes the set of the eight neighbours in cell *c*’s neighbourhood domain. Hence, in the present work, the maximum value of ${s}_{c}^{n}(t)$ is 8, and the minimum value is 0. This local information allows an agent to identify which of the four adjacent cells are less at risk (surrounded by more neighbours) such that it can make movement decisions based on the relative positions of neighbours.

### Behavioural Genotypes

The movement strategy, i.e., the causal mapping from each agent’s locally perceived information to its direction choice, was implemented by a two-layer fully connected artificial neural network, which was composed of 11 input nodes and 4 output nodes (Fig. 5). Regarding the input nodes, eight nodes delivered the occupancy states, ${s}_{c}^{o}(t)$, and neighbourhood sizes, ${s}_{c}^{n}(t)$, of the four adjacent cells. Another node was used to store the neighbourhood size of the focal cell. All the neighbourhood sizes were divided by 8.0 to normalise the values between 0.0 and 1.0. We used an input node providing a random floating-point value uniformly distributed between 0.0 and 1.0 to allow the development of mixed strategies if necessary. Lastly, a node that delivered a constant value of 1.0 was added as the bias unit.

The weights of all links in each neural network were randomly generated in the first generation and then inherited at reproduction, including a mutation operator (see Methods, Evolutionary Procedure). Once the values of the input nodes had been assigned, the values of the four output nodes were updated. These nodes represented the directions to the north, south, east and west of the focal agent. The final direction was triggered by the node with the largest output value.

### Evolutionary Procedure

Following the selfish herd scenario^1,16^, we assumed that the predation risk of an agent was negatively correlated to the average number of neighbours during its movements over the whole course of a generation. We defined the neighbourhood score of each agent *i*, ${S}_{i}^{N}$, to evaluate the mean neighbourhood size per time step by

2 $${S}_{i}^{N}=\frac{1}{T}\,\sum _{t=1}^{T}\,{s}_{{x}_{i}(t)}^{n}(t),$$

where *x*_*i*_(*t*) records the occupied cell by agent *i* at time step *t*, and $T=\mathrm{2,000}$ is the number of total time steps in each generation. The agents with low neighbourhood scores are at more risk within the generation.

Our evolutionary process followed the common evolutionary procedure in artificial life models^18–20^. After each individual’s neighbourhood score, ${S}_{i}^{N}$, was evaluated through Eq. (2), the *μ* proportion of the population that received the lowest ${S}_{i}^{N}$ was assumed to be killed by predation, where *μ* was set to a rate of 0.05 per generation. Assuming that the environment had a stable carrying capacity to support a certain number of prey agents in the long term, we then maintained this population size by adding the same number of offspring agents as were predated before. Each offspring agent was reproduced from two uniformly randomly selected agents out of all survivors, i.e., the truncation selection scheme. During reproduction, the weight of each link in the offspring neural network inherited one of its parents at random, with a mutation rate of $\varepsilon \sim N(0,0.0005)$, which rate was strong enough to generate potentially beneficial mutants within reasonably many generations (see Methods, Parameter Scans). The survivors and offspring agents then entered the next generation and repeated the procedure until the 3,000^th^ generation had been attained.

### Measurements

To quantify the degree of movement activity of agents^6^, the mobility score of each agent *i* was defined by

3 $${S}_{i}^{M}=\frac{1}{T}\,\sum _{t=1}^{T}\,\phi ({x}_{i}(t),{x}_{i}(t+1)),\,{\rm{where}}\,\phi (a,b)=\{\begin{array}{ll}0, & {\rm{if}}\,a=b\\ 1, & {\rm{otherwise}}\,\end{array}$$

as the frequency of moving from the present cell to an adjacent empty cell per time step, i.e., ${x}_{i}(t)\ne {x}_{i}(t+1)$, where *x*_*i*_(*t*) and ${x}_{i}(t+1)$ are agent *i*’s locations at time step *t* and time step $t+1$, respectively. In addition, the neighbourhood score of an agent, defined in Eq. (2), was used to measure the social inclusion exhibited by individuals^6^.

To further identify the features of individual movement behaviours (as behavioural phenotypes), we recorded the probability of agent *i* moving to a vacant cell under the condition of the present cell’s neighbourhood size, i.e.,

4 $$pr({x}_{i}(t)\ne {x}_{i}(t+1)|{s}_{{x}_{i}(t)}^{n}(t)),$$

where ${s}_{{x}_{i}(t)}^{n}(t)$ is the number of neighbours around agent *i* at time step *t* as defined in Eq. (1). Lastly, at the genotype level, the likelihood of agent *i* and agent *j*’s strategies, denoted by $L(i,j)$, was defined as one minus the mean difference of the link weights between their neural networks:

5 $$L(i,j)=1-\frac{1}{44}\,\sum _{I=1}^{11}\,\sum _{O=1}^{4}\,|{w}_{i}^{I,O}-{w}_{j}^{I,O}|,$$

where *I* indexes all eleven input nodes, *O* indexes all four output nodes, and ${w}_{i}^{I,O}$ represents the weight of agent *i*’s link from node *I* to node *O*.

### Parameter Scans

In the present model, we used the simplest selection scheme, i.e., the truncation selection, for the evolutionary procedure, which reflects the selection of herd animals in nature. Other popular selection schemes, such as tournament selections or roulette-wheel selections, are also practical from an evolutionary computation perspective. The proportion of killed agents, *μ*, is insensitive to the final emergent patterns, but the phase transitions in evolution may be less clear with a large *μ*. The mutation rate of each link weight was set to $\varepsilon \sim N(0,0.0005)$, which implies that the probability an offspring network receiving a considerable weight mutation (greater than 10% of the average absolute weight value, which was approximately 0.7) from at least one of its 44 links is 10% and is strong enough to cover most potential strategies in 3,000 generations. We suggest this rate should not exceed $\varepsilon \sim N(0,0.001)$ for demonstration robustness.

Regarding the comprehensiveness of the proposed model, the choice to evaluate neighbourhood scores by the Moore neighbourhood with radius $r=1$ was to minimise the computational time. The simulation outputs were consistent given a larger neighbourhood domain, such as the Moore neighbourhood with radius 2–4. Additionally, the usage of two-layer neural networks is a simplification from our preliminary work, where a neuro-evolutionary algorithm was introduced^45^. We found that under the proposed environment, a much simpler network structure and selective mechanism were sufficient to capture all evolutionary features in comparable quality. Hence, we propose a model that focuses on the influence of crowdedness by incorporating a minimum of factors and maintaining all relevant aspects.

## Supplementary information

SIGuide

Supplementary Video S1

Supplementary Video S2
