# Supplementary material for: Collective Motion as an Ultimate Effect in Crowded Selfish Herds
Source: Sci Rep. 2019 Apr 29;9:6618. doi: 10.1038/s41598-019-43179-6 (PMC6488663; doi:10.1038/s41598-019-43179-6)
Supplement: Supplementary file 1 — SIGuide [file 41598_2019_43179_MOESM1_ESM.doc]

**Collective Motion as an Ultimate Effect in Crowded Selfish Herds**

**Wen-Chi Yang**^1,*^ **and Thomas Schmickl**^2^

^1^Department of Computer Science and Technology, Henan Institute of Technology, Xinxiang 453003, China

^2^Artificial Life Lab of the Institute of Biology, Karl-Franzens University of Graz, A-8010 Graz, Austria

*w.yang@hait.edu.cn

**Supplementary Information**

Supplementary Video S1

Video S1.mp4 (3.5 MB)

Caption:

An evolutionary simulation with the crowding effect. When the egoistic agents were forbidden from entering a crowded herd, they evolved into mobile herds through the attempt of border individuals to shift along the edge and share predation risk with the inner neighbours.

Supplementary Video S2

Video S2.mp4 (3.4 MB)

Caption:

An evolutionary simulation without the crowding effect. When the egoistic agents had no difficulty to enter a crowded herd, they evolved into stationary herds through the attempt of border individuals to squeeze into the group Interior.
